# L-Arginine Supplementation Improves Endurance Under Chronic Fatigue: Inducing In Vivo Paradigms with In Vitro Support

**DOI:** 10.3390/nu17203239

**Published:** 2025-10-15

**Authors:** Somin Lee, Woo Nam, Kyu Sup An, Eun-Ji Cho, Yong-Min Choi, Hyeon Yeol Ryu

**Affiliations:** 1 Korea Conformity Laboratories, 8, Gaetbeol-ro 145 Beon-gil, Yeonsu-gu, Incheon 21999, Republic of Korea; somin14@kcl.re.kr (S.L.); nw89@kcl.re.kr (W.N.); ksahn@kcl.re.kr (K.S.A.); 2 Daesang Wellife, Seoul 03130, Republic of Korea; eunjicho@daesang.com (E.-J.C.); ymchoi@daesang.com (Y.-M.C.)

**Keywords:** L-arginine, anti-fatigue, energy metabolism, oxidative stress, C2C12 myoblasts

## Abstract

Background: L-arginine is a conditionally essential amino acid that serves as a substrate for nitric oxide synthase and regulates energy metabolism. While its ergogenic effects have been proposed, the mechanisms underlying its anti-fatigue properties are not fully understood. Methods: Male ICR mice were orally administered L-arginine (300, 600, or 1200 mg/kg bw/day) for 28 days. Fatigue was chronically induced using twice-weekly forced swimming or treadmill running, and fatigue resistance was then assessed under these paradigms. Blood, skeletal muscle, and liver were analyzed for biomarkers including glucose, lactate, LDH, CPK, NEFA, ammonia, glycogen, nitric oxide, cortisol, and antioxidant enzymes. In parallel, C2C12 myoblasts were treated with L-arginine under proliferative and differentiated conditions to assess hexokinase (HK) activity, myogenin expression, and ROS generation. Results: In vivo, L-arginine decreased serum LDH, CPK, NEFA, ammonia, nitric oxide, and cortisol, while enhancing blood glucose and glycogen storage in both muscle and liver. Forced swimming reduced serum lactate, whereas treadmill exercise elevated intramuscular lactate, suggesting context-dependent lactate regulation. Importantly, L-arginine did not significantly improve forced-swimming immobility time, whereas treadmill time-to-exhaustion increased at the highest dose. Antioxidant responses were improved, as reflected by normalized hepatic catalase activity. In vitro, L-arginine increased HK activity, promoted myogenin expression, and reduced ROS levels, supporting improved glucose utilization, muscle differentiation, and oxidative stress resistance. Conclusions: These findings demonstrate that L-arginine supplementation under chronic fatigue-inducing paradigms improves endurance and alleviates fatigue by enhancing energy metabolism, preserving glycogen, reducing muscle injury, and attenuating oxidative stress. L-arginine shows potential as a functional ingredient for promoting exercise performance and recovery.

## 1. Introduction

Fatigue, particularly exercise-induced fatigue, is a multifactorial condition characterized by reduced physical performance, accumulation of metabolic byproducts, and oxidative stress [1,2]. Nutritional interventions have gained attention as safe and practical strategies to mitigate fatigue and improve exercise capacity. Among functional nutrients, amino acids play pivotal roles in energy metabolism, muscle physiology, and recovery from exercise stress [3].

L-arginine is a conditionally essential amino acid that functions as a substrate for nitric oxide synthase (NOS) and participates in the urea cycle, thereby influencing vascular tone, ammonia detoxification, and metabolic regulation [4]. Previous studies have suggested that L-arginine supplementation may improve endurance performance, enhance oxygen delivery, and modulate immune and antioxidant responses [5,6]. L-arginine has been reported to increase glycogen storage, regulate lactate metabolism, and attenuate markers of muscle injury in animal models and human trials [7]. However, despite these promising observations, the evidence remains inconsistent, and the mechanistic insights are limited.

Most available studies have focused either on physiological outcomes in vivo or on isolated biochemical effects in vitro, with relatively few investigations integrating both approaches. Furthermore, the role of L-arginine in regulating oxidative stress, muscle metabolism, and fatigue-related biomarkers across different experimental models remains to be clarified.

Several physiological determinants link L-arginine to exercise performance, including nitric-oxide-dependent perfusion and oxygen delivery, ammonia detoxification via the urea cycle, and preservation of glucose homeostasis and glycogen stores [4,5,6,7,8,9,10]. Moreover, contemporary concepts of lactate as an oxidative fuel and signaling molecule suggest that the impact of L-arginine on fatigue may depend on exercise intensity and oxygen availability [11,12,13]. Building on these considerations, we focused on functional outcomes (endurance and fatigue-related biomarkers) together with cellular readouts of glucose metabolism, myogenic differentiation, and oxidative stress to provide convergent evidence across in vivo and in vitro models.

Therefore, this study aimed to evaluate the anti-fatigue efficacy of L-arginine using both in vivo and in vitro approaches. Specifically, forced swimming and treadmill exercise tests in ICR mice were employed to assess endurance performance and biochemical markers in blood, muscle, and liver. In parallel, C2C12 myoblasts were used to examine the effects of L-arginine on glucose metabolism, muscle differentiation, and oxidative stress. By combining animal and cellular models, this study sought to provide comprehensive evidence supporting the potential of L-arginine as a functional ingredient to improve exercise performance and recovery.

## 2. Materials and Methods

### 2.1. In vivo Experiments

#### 2.1.1. Animals and Experimental Design

Male ICR mice (7 weeks old) were purchased from OrientBio, Gyeonggi-do, Korea and acclimated for 1 week under controlled conditions (22 ± 2 °C, 55 ± 10% humidity, 12 h light/dark cycle) with ad libitum access to food and water. Animals were sorted by weight, and the average weight of all groups was adjusted to minimize intergroup variation. Cage position and testing order were balanced across groups to minimize potential confounders. The FST and treadmill experiments were conducted in separate cohorts, with the same group allocation and animal care. The group size was n = 8 per group, based on prior studies and logistical considerations. Since the purpose of this animal experiment was to determine fatigue improvement, we selected the maximum number of animals that could be cared for and tested simultaneously. We also specified a reliable number of animals. Male ICR mice were randomly assigned to five groups: control, negative control, and L-arginine treatment groups (300, 600, and 1200 mg/kg bw/day), given via oral gavage once daily for 28 days. L-arginine was dissolved in sterile distilled water. Based on weekly body weights, the dosing volume was 10 mL/kg bw/day. The control and model control groups were gavaged with the vehicle at the same volume. Dose selection was based on allometric conversion from the intended human intake (50 mg/kg bw/day), yielding a mouse equivalent of ≈617 mg/kg/day; thus, 600 mg/kg/day was chosen with 0.5× and 2× doses to assess dose–response. Throughout the study period, all animals were observed once daily for general clinical symptoms, including mortality, appearance of abnormal behaviors, and onset or severity of any visible signs. All procedures were conducted in accordance with institutional animal care guidelines, and clinical signs and body weights were monitored weekly. Forced swimming and treadmill experiments were conducted in independent cohorts with identical group allocation (*n* = 8 per group in each cohort; total *n* = 80). Animals were monitored at least once daily by trained personnel. Humane endpoints were predefined (≥20% body-weight loss, severe lethargy, or self-injury); none were reached. The study followed the 3Rs: replacement was not applicable to the physiological endpoints, reduction was addressed by using the minimum sample size consistent with statistical detectability, and refinement was implemented via environmental control and humane monitoring.

#### 2.1.2. Forced-Swimming Test (FST)

To assess endurance, mice were individually placed in a transparent cylindrical tank (10 cm diameter, 25 cm height) filled with water (15 cm depth, 23 ± 2 °C). Animals were subjected to forced swimming for 6 min twice per week during the treatment period. On the final day, 30 min after administration, mice were allowed to swim for 6 min. Immobility time during the last 4 min was recorded, following a 2 min acclimation period. Immobility was defined as the absence of active swimming except minimal movements to keep the head above water.

#### 2.1.3. Treadmill Exercise Test

Treadmill running was used as a fatigue-induction protocol rather than an endurance training regimen in an independent cohort. Exhaustion was defined a priori as the inability to resume running pace after gentle encouragement and remaining on the platform for ≥10 s (three consecutive prompts). Mice were preconditioned twice per week with incremental speeds (10 m/min for 10 min, 16 m/min for 10 min, and 21 m/min for 10 min: total 30 min). On the final day, endurance was evaluated by running at 10 m/min for 10 min, 16 m/min for 3 min, 21 m/min for 3 min, and finally 40 m/min for up to 30 min. The total exercise time until exhaustion was recorded for each animal.

#### 2.1.4. Sample Collection and Biochemical Analyses

Following exercise tests, animals were euthanized with isoflurane, and blood was collected via abdominal aorta puncture. Liver and soleus muscle were excised, rinsed, and homogenized. Plasma and tissue homogenates were analyzed for biomarkers of fatigue using an automated biochemical analyzer (Hitachi 3500, HITACHI, Tokyo, Japan) and commercial kits: glucose (GLU, glycogen: glycogen assay kit, ab65620, Abcam, Waltham, MA, USA), lactate (LAC), LDH, CPK, NEFA, nitric oxide (NO), ammonia, glycogen, cortisol, superoxide dismutase (SOD, Colorimetric Activity Kit, EIASODC, Thermo-Fisher Scientific, Fredrick, MD, USA), and catalase. Muscle and liver glycogen were quantified using a glycogen assay kit (ab65620, Abcam, Cambridge, UK). Plasma and tissue nitric oxide was quantified as total NOx (nitrite/nitrate) using a nitrate reductase–coupled Griess colorimetric method according to the manufacturer’s instructions.

### 2.2. In Vitro Experiments

#### 2.2.1. Cell Culture and Differentiation

C2C12 mouse myoblasts, obtained from the American Type Culture Collection (ATCC, Manassas, VA, USA) with catalog number CRL-1772 were cultured in Dulbecco’s Modified Eagle’s Medium (DMEM; Gibco, Grand Island, NY, USA) supplemented with 10% fetal bovine serum (FBS) and 1% penicillin–streptomycin at 37 °C in a humidified atmosphere with 5% CO_2_. For differentiation, cells were seeded at 2.0 × 10^5^ cells/well in 6-well plates and, upon reaching 90% confluence, switched to DMEM containing 2% horse serum without antibiotics for 7 days with medium changes every 2 days.

#### 2.2.2. Treatments

Cells were treated with L-arginine (100, 250, or 500 μg/mL) for 24 h. Oxidative stress was induced using hydrogen peroxide (H_2_O_2_, 300 μM for viability assay, 4 mM for oxidative stress assay). Treatment concentrations and durations were determined based on prior cell viability tests, and all treatments were conducted for 24 h.

#### 2.2.3. Cell Viability Assay

Cell viability was determined in undifferentiated C2C12 cells using the CCK-8 assay (Dojindo, Japan). Cells were seeded in 96-well plates (1.0 × 10^4^ cells/well), treated with L-arginine and/or H_2_O_2_ for 24 h, incubated with CCK-8 reagent for 1 h, and absorbance was measured at 450 nm.

#### 2.2.4. Oxidative Stress (ROS) Measurement

Intracellular ROS was measured using 2′,7′-dichlorodihydrofluorescein diacetate (H_2_DCF-DA; Invitrogen, Carlsbad, CA, USA). Cells were incubated with 10 μM H_2_DCF-DA for 30 min, washed, and exposed to H_2_O_2_ (1 mM) with or without L-arginine for 30 min. Fluorescence intensity was measured at 485/530 nm using a microplate reader (SpectraMax iD3, Molecular Devices, San Jose, CA, USA).

#### 2.2.5. Metabolic and Myogenic Markers

Cell lysates were prepared with RIPA buffer containing protease inhibitor cocktail. Hexokinase (HK) activity was determined using a commercial assay kit (Sigma-Aldrich, Burlington, MA, USA). Myogenin expression was quantified using a mouse myogenin ELISA kit (LifeSpan BioSciences, Seattle, WA, USA), according to the manufacturer’s instructions. Protein concentrations were normalized prior to analysis.

### 2.3. Statistical Analysis

Data are expressed as mean ± standard deviation (SD). Normality and homoscedasticity were assessed using the Shapiro–Wilk and Levene tests, respectively. For comparisons involving ≥3 groups, one-way ANOVA was applied, followed—when assumptions were met—by Dunnett’s post hoc test versus the model control. If homoscedasticity was violated, Dunnett’s T3 procedure was used for multiple comparisons versus the model control. Two-group comparisons were performed only when required using independent-sample *t*-tests. Two-sided *p* < 0.05 was considered statistically significant. All statistical analyses were conducted using SPSS version 12.0K (SPSS Inc., Chicago, IL, USA) in accordance with the institutional standard operating procedures for data processing. All experimental data were processed according to the company’s standard operating procedures, and prior inclusion/exclusion criteria were defined (illness or injury, technical failure, or extreme outliers > 3 SD). No animals or data points were excluded unless otherwise stated. The exact n value for each analysis is reported in the figure legends.

## 3. Results

### 3.1. Forced-Swimming Test

#### 3.1.1. Endurance Performance

**Figure 1:**
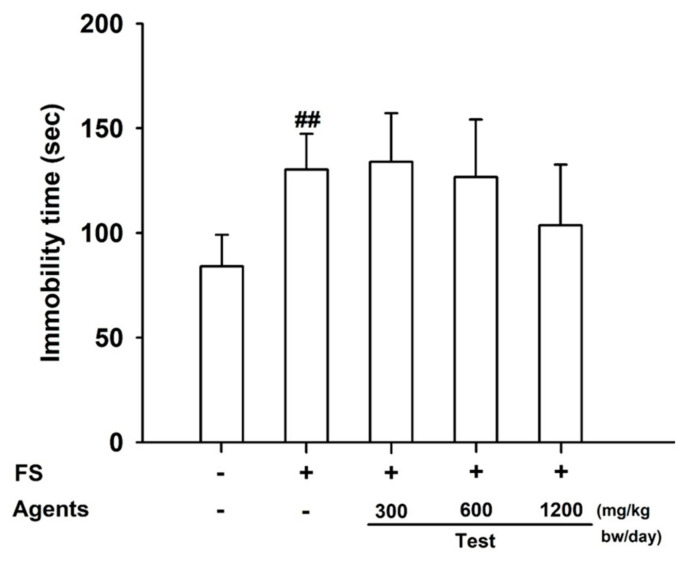
Effects of L-arginine on endurance in the forced-swimming test. Data are presented as mean ± SD (*n* = 8 per group) ##: *p* < 0.01, vs. control group (Student’s *t*-test). The symbols ‘+’ and ‘−’ indicate the presence and absence of the specific experimental condition, respectively.

The forced-swimming test revealed that immobility time was significantly increased in the model control group compared with the control (*p* < 0.01), confirming successful fatigue induction. Between-group differences did not reach statistical significance; however, the 1200 mg/kg group tended to show lower immobility time than the model control (trend; *p* = 0.169), consistent with a dose-related tendency toward reduced immobility (Figure 1).

#### 3.1.2. Serum Biochemical Parameters

##### Markers of Muscle Injury

**Figure 2:**
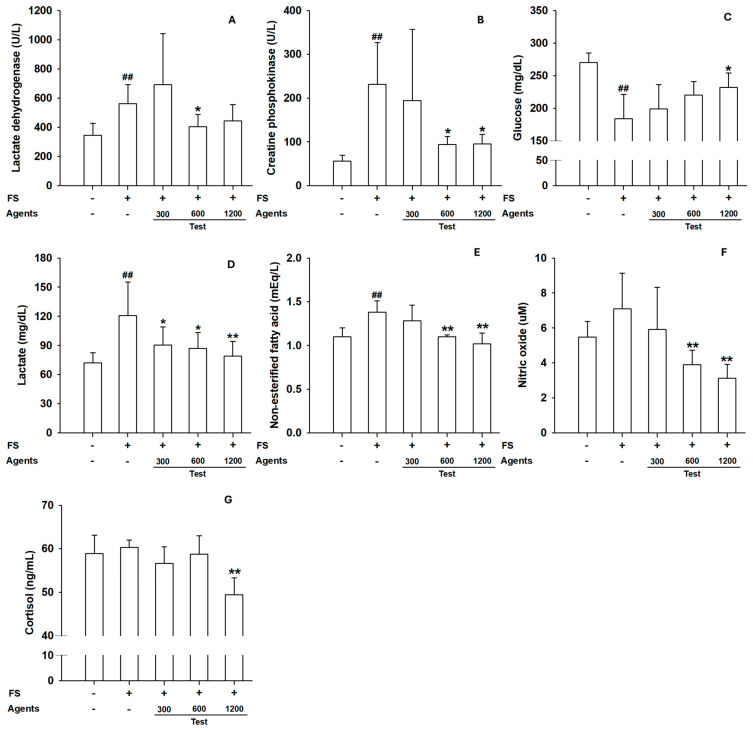
Effects of L-arginine on serum biochemical parameters in the forced-swimming test. (**A**) Serum lactate dehydrogenase, (**B**) Creatine phosphokinase, (**C**) Glucose, (**D**) Lactate, (**E**) Non-esterified fatty acid, (**F**) Nitric oxide, (**G**) Cortisol levels. Data are presented as mean ± SD (*n* = 8 per group). ## *p* < 0.01 vs. control group (Student’s *t*-test); * *p* < 0.05, ** *p* < 0.01 vs. model control group (one-way ANOVA with Dunnett or Dunnett T3 if variances were unequal). The symbols ‘+’ and ‘−’ indicate the presence and absence of the specific experimental condition, respectively.

Markers of muscle injury, including serum LDH and CPK, were significantly elevated in the model control group compared with the control (*p* < 0.01). L-arginine supplementation at 600 and 1200 mg/kg bw/day reduced LDH and CPK levels, suggesting potential attenuation of muscle damage (Figure 2A,B). In addition, serum lactate levels decreased across all L-arginine groups, supporting improved fatigue resistance.

##### Other Fatigue-Related Biomarkers

Other biomarkers showed dose-dependent improvements. Blood glucose levels were significantly increased in the 1200 mg/kg groups, whereas NEFA levels decreased at ≥600 mg/kg compared with the model control. Serum nitric oxide levels were reduced at ≥600 mg/kg, and cortisol levels decreased at 1200 mg/kg, indicating reduced metabolic and stress burden under exercise load (Figure 2C–G).

#### 3.1.3. Tissue Analyses

**Figure 3:**
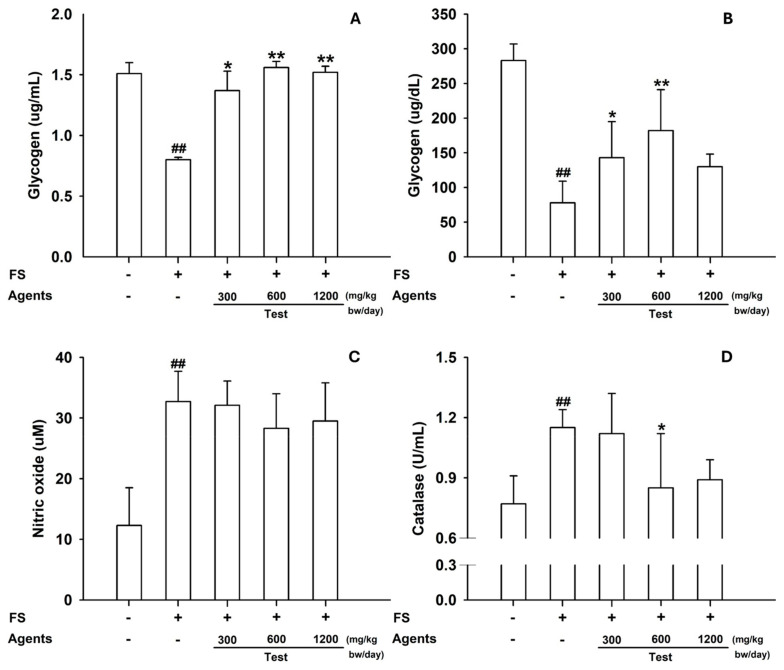
Tissue biomarkers in the forced-swimming test. (**A**) Skeletal muscle glycogen content. (**B**) Hepatic glycogen, (**C**) NO, and (**D**) Catalase. Data are presented as mean ± SD (*n* = 8 per group). ##: *p* < 0.01 vs. control group (Student’s *t*-test); * *p* < 0.05, ** *p* < 0.01 vs. model control group (one-way ANOVA with Dunnett or Dunnett T3 if variances were unequal). The symbols ‘+’ and ‘−’ indicate the presence and absence of the specific experimental condition, respectively.

Skeletal muscle glycogen was significantly reduced in the model control, but restored by L-arginine (*p* < 0.05 at 300 mg/kg; *p* < 0.01 at ≥600 mg/kg) (Figure 3A). In the liver, glycogen content increased across all L-arginine doses, whereas hepatic catalase—elevated in the model control—was reduced at 600 mg/kg (*p* < 0.05) and showed a similar decreasing trend at 1200 mg/kg (Figure 3B–D).

### 3.2. Treadmill Exercise Test

#### 3.2.1. Endurance Performance

**Figure 4:**
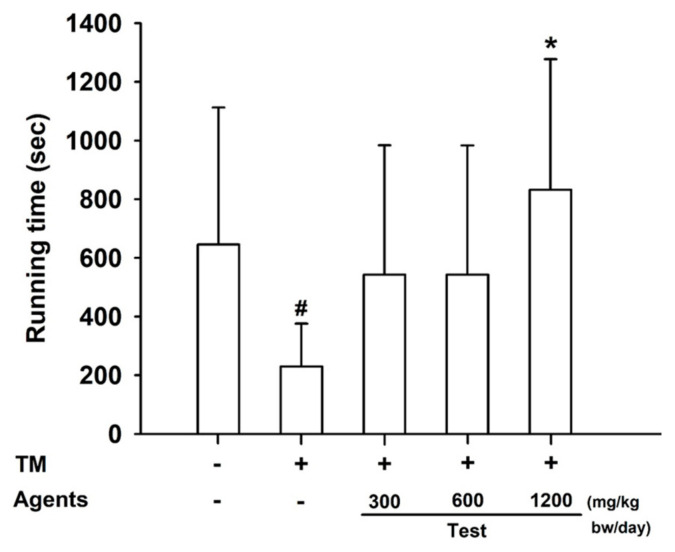
Effects of L-arginine on treadmill endurance test. Data are presented as mean ± SD (*n* = 8 per group). #: *p* < 0.05 vs. control group (Student’s *t*-test); * *p* < 0.05 vs. model control group (one-way ANOVA with Dunnett or Dunnett T3 if variances were unequal). The symbols ‘+’ and ‘−’ indicate the presence and absence of the specific experimental condition, respectively.

In the treadmill test, mice receiving L-arginine at 1200 mg/kg bw/day exhibited a significant increase in exercise duration compared with both control and model control groups (*p* < 0.05), demonstrating enhanced endurance capacity (Figure 4).

#### 3.2.2. Serum Biochemical Parameters

**Figure 5:**
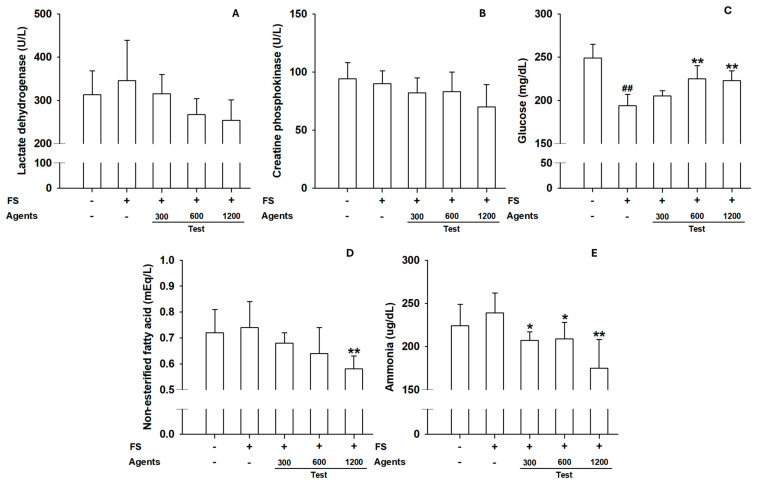
Effects of L-arginine on serum biochemical parameters in treadmill endurance test. (**A**) Serum lactate dehydrogenase, (**B**) Creatine phosphokinase, (**C**) Glucose, (**D**) Non-esterified fatty acid, (**E**) Ammonia levels. Data are presented as mean ± SD (*n* = 8 per group). ##: *p* < 0.01 vs. control group (Student’s *t*-test); * *p* < 0.05, ** *p* < 0.01 vs. model control group (one-way ANOVA with Dunnett or Dunnett T3 if variances were unequal). The symbols ‘+’ and ‘−’ indicate the presence and absence of the specific experimental condition, respectively.

Although not statistically significant, LDH and CPK levels tended to decrease in L-arginine groups, suggesting protection against muscle injury (Figure 5A,B). Blood glucose increased at ≥600 mg/kg. NEFA levels decreased significantly at 1200 mg/kg, consistent with improved energy utilization. Ammonia levels were significantly reduced in groups receiving at 1200 mg/kg (Figure 5C–E), indicating reduced metabolic waste under sustained exercise.

#### 3.2.3. Tissue Analyses

**Figure 6:**
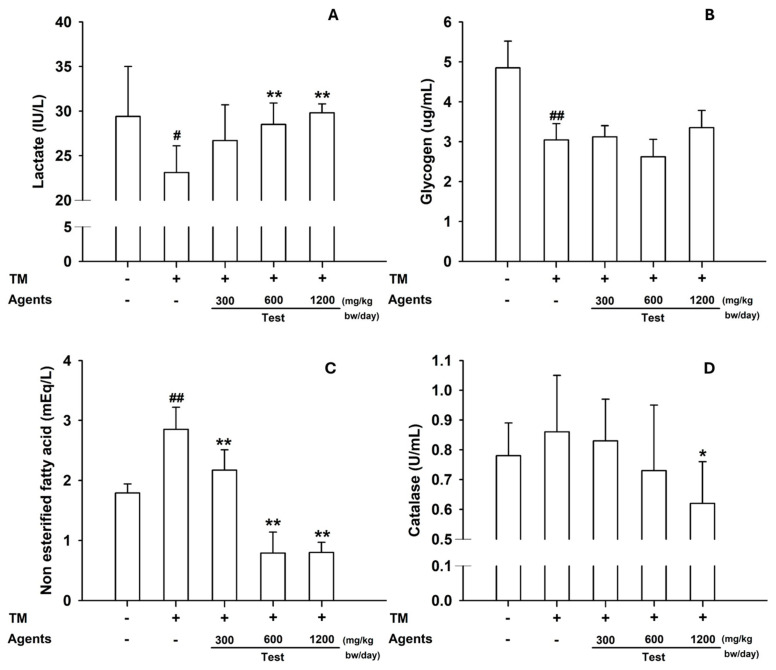
Tissue biomarkers in the treadmill endurance test. (**A**) Skeletal muscle lactate and (**B**) Glycogen content. (**C**) Hepatic NEFA, and (**D**) Catalase levels. Data are presented as mean ± SD (*n* = 8 per group). #: *p* < 0.05, ##: *p* < 0.01 vs. negative control group (Student’s *t*-test); * *p* < 0.05, ** *p* < 0.01 vs. control group (one-way ANOVA with Dunnett or Dunnett T3 if variances were unequal). The symbols ‘+’ and ‘−’ indicate the presence and absence of the specific experimental condition, respectively.

In skeletal muscle, lactate content was increased in mice treated with L-arginine at ≥600 mg/kg, reflecting its role as an energy substrate and signaling metabolite during exercise (Figure 6A). Muscle glycogen content was also slightly elevated, particularly at 1200 mg/kg. In the liver, NEFA decreased significantly at ≥300 mg/kg, and catalase activity was reduced at 1200 mg/kg (Figure 6B–D), suggesting lower oxidative stress compared with the model control.

### 3.3. In Vitro Assays with C2C12 Cells

#### 3.3.1. Cell Viability

**Figure 7:**
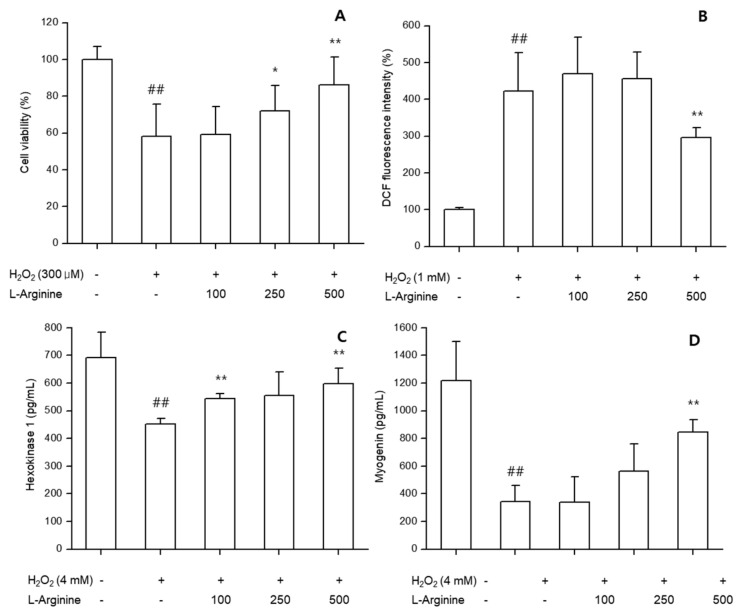
In vitro effects of L-arginine on C2C12 cells. (**A**) Cell viability after H_2_O_2_ challenge. (**B**) ROS production in undifferentiated myoblasts. (**C**) Hexokinase (HK) activity in differentiated myotubes. (**D**) Myogenin expression in differentiated myotubes. Data are presented as mean ± SD of at least three independent experiments. ##: *p* < 0.01 vs. negative control group (Student’s *t*-test); * *p* < 0.05, ** *p* < 0.01 vs. control group (one-way ANOVA with Dunnett or Dunnett T3 if variances were unequal). The symbols ‘+’ and ‘−’ indicate the presence and absence of the specific experimental condition, respectively.

L-arginine treatment protected undifferentiated C2C12 myoblasts against H_2_O_2_-induced cytotoxicity. Cell viability was significantly higher in L-arginine groups (100–500 μg/mL) compared with H_2_O_2_-only treatment (*p* < 0.05), indicating cytoprotective effects (Figure 7A).

#### 3.3.2. Oxidative Stress (ROS)

In undifferentiated cells, L-arginine significantly reduced intracellular ROS levels induced by H_2_O_2_, in a concentration-dependent manner (*p* < 0.01) (Figure 7B).

#### 3.3.3. Metabolic and Myogenic Markers

In differentiated C2C12 cells, L-arginine significantly enhanced HK activity compared with controls, suggesting improved glucose utilization. Myogenin expression was also increased in L-arginine-treated groups, reflecting promotion of myogenic differentiation (Figure 7C,D).

## 4. Discussion

L-arginine supplementation exerted anti-fatigue effects across complementary models. In C2C12 myoblasts, L-arginine enhanced glucose metabolism, promoted myogenic differentiation, and reduced oxidative stress, as reflected by increased hexokinase activity, upregulated myogenin, and suppressed ROS. These cellular changes are consistent with improved muscle glucose handling and differentiation capacity, both of which are mechanistically linked to performance and recovery [8,9].

The in vitro findings aligned with in vivo outcomes in ICR mice subjected to forced swimming and treadmill running. Improved glycogen repletion in skeletal muscle and liver coheres with enhanced glucose phosphorylation capacity, whereas reductions in circulating creatine kinase and lactate dehydrogenase support attenuation of exercise-induced muscle damage [10,14,15].

Lactate metabolism appeared context-dependent. In the forced-swimming test, L-arginine lowered serum lactate, suggesting reduced reliance on anaerobic glycolysis; in the treadmill test, intramuscular lactate rose, compatible with lactate’s recognized roles as an oxidative fuel and signaling molecule that can facilitate recovery and adaptation [11,12,13]. Repeated forced swimming constitutes a substantial stressor and can promote fatigability in control animals; thus, our forced-swimming test paradigm evaluated anti-fatigue efficacy under stress-induced fatigue conditions. Within this context, L-arginine’s trends toward attenuated serum lactate and favorable modulation of hepatic catalase and cortisol are consistent with improved stress coping and metabolic efficiency under load.

Compared with previous studies, the present findings suggest that L-arginine may enhance metabolic efficiency under aerobic exercise conditions, thereby attenuating excessive lactate accumulation [16,17]. This supports the potential application of L-arginine supplementation as a strategy to improve exercise performance and reduce fatigue. Moreover, under anaerobic exercise conditions, L-arginine may contribute to performance via complementary mechanisms, including improved blood flow through nitric-oxide-dependent vasodilation and facilitation of glycogen repletion [18,19]. Taken together, the metabolic effects of L-arginine likely depend on exercise intensity, oxygen availability, and experimental models, warranting further research across diverse protocols to clarify underlying mechanisms.

Although NO signaling was not assayed in vitro, elevated serum NO in vivo is congruent with L-arginine’s role as a nitric-oxide synthase substrate, potentially improving perfusion and O_2_ delivery during exercise [20,21]. Follow-up cell work interrogating eNOS/nNOS activation and NO/cGMP readouts would clarify this link. Likewise, profiling arginase and urea-cycle enzymes could mechanistically explain the observed reduction in blood ammonia during treadmill exercise [22].

Collectively, the data indicate that L-arginine improves endurance and mitigates fatigue via convergent mechanisms—enhanced glucose utilization, preservation of glycogen stores, reduction in muscle injury, context-appropriate modulation of lactate, and dampening of oxidative stress—supporting its candidacy as a functional ingredient for performance and recovery.

### Limitations and Future Directions

Our in vitro panel was limited to HK, myogenin, and ROS; broader pathway coverage (e.g., NO/cGMP, mitochondrial biogenesis, and urea-cycle flux) is warranted. Translation from rodents and immortalized cells to humans remains to be established with dose–response and regimen optimization. Future studies should integrate metabolic and molecular endpoints—MCT1/MCT4 and lactate shuttling, mitochondrial biogenesis (PGC-1α), fatty-acid oxidation control (CPT1b), and glycogen synthase regulation—to map L-arginine’s system-level effects on exercise metabolism [23,24,25,26,27,28,29].

## 5. Conclusions

In summary, this study demonstrated that L-arginine supplementation improves exercise endurance and mitigates fatigue through coordinated effects on energy metabolism, muscle protection, and oxidative stress regulation.

In vivo, endurance improvement was evident under the treadmill paradigm—with increased time-to-exhaustion at the highest dose—whereas the forced-swimming immobility time did not show a statistically significant improvement. Across both paradigms, these functional outcomes were accompanied by favorable modulation of biochemical markers, including glucose, glycogen, lactate, LDH, CPK, NEFA, ammonia, nitric oxide, and antioxidant enzymes. Complementary in vitro assays in C2C12 myoblasts confirmed that L-arginine promotes glucose utilization, supports myogenic differentiation, and reduces ROS generation, thereby providing cellular evidence for its anti-fatigue effects. These findings suggest that L-arginine has potential as a functional ingredient to enhance exercise performance and recovery, while its effects on behavioral endpoints may be model-dependent. Nonetheless, further studies are needed to elucidate detailed molecular pathways, validate efficacy in human subjects, and establish optimal dosing strategies for practical applications.

## Data Availability

All relevant data are contained within the article. Additional raw data are available from the corresponding author upon reasonable request.

## Abbreviations

The following abbreviations are used in this manuscript:

ATCC	American Type Culture Collection
CCK-8	Cell counting kit-8
cGMP	Cyclic guanosine monophosphate
CPK	Creatine phosphokinase
CPT1b	Carnitine O-palmitoyltransferase 1B
DMEM	Dulbecco’s Modified Eagle’s Medium
FBS	fetal bovine serum
GLU	Glucose
HK	Hexokinase
IACUC	the Institutional Animal Care and Use Committee
ICR	Institute of Cancer Research
LAC	Lactate
LDH	Lactate dehydrogenase
MCT	Monocarboxylate transporter
NEFA	Non-esterified fatty acid
NOS	Nitric oxide synthase
PGC-1α	Peroxisome proliferator-activated receptor-γ coactivator 1α
ROS	Reactive oxygen species
SD	Standard deviation
SOD	Superoxide dismutase
